# Recombinant pseudorabies virus with gI/gE deletion generated by overlapping polymerase chain reaction and homologous recombination technology induces protection against the PRV variant PRV-GD2013

**DOI:** 10.1186/s12917-021-02861-6

**Published:** 2021-04-14

**Authors:** Wenhui Li, Dijing Zhuang, Hong Li, Mengpo Zhao, Erpeng Zhu, Baoming Xie, Jinding Chen, Mingqiu Zhao

**Affiliations:** 1 College of Veterinary Medicine, South China Agricultural University, 483 Wu Shan Road, Tianhe District, Guangzhou, 510642 Guangdong Province China; 2 Guangdong Laboratory for Lingnan Modern Agriculture, Guangzhou, China; 3 Shandong Qianxi Agriculture & Animal Husbandry Development Co., Ltd., Zaozhuang, China

**Keywords:** Pseudorabies virus, Recombinant virus, PRV-GD2013-ΔgI/gE, Vaccine

## Abstract

**Background:**

Since 2011, numerous highly virulent and antigenic variant viral strains have been reported in pigs that were vaccinated against the swine pseudorabies virus. These infections have led to substantial economic losses in the Chinese swine industry.

**Results:**

This study, constructed a novel recombinant vaccine strain with gI/gE deletion (PRV-GD2013-ΔgI/gE) by overlapping PCR and homologous recombination technology. The growth curves and plaque morphology of the recombinant virus were similar to those of the parental strain. However, PRV-GD2013-ΔgI/gE infection was significantly attenuated in mice compared with that of PRV-GD2013. Two-week-old piglets had normal rectal temperatures and displayed no clinical symptoms after being inoculated with 10^5^ TCID_50_ PRV-GD2013-ΔgI/gE, indicating that the recombinant virus was avirulent in piglets. Piglets were immunized with different doses of PRV-GD2013-ΔgI/gE, or a single dose of Bartha-K61 or DMEM, and infected with PRV-GD2013 at 14 days post-vaccination. Piglets given high doses of PRV-GD2013-ΔgI/gE showed no obvious clinical symptoms, and their antibody levels were higher than those of other groups, indicating that the piglets were completely protected from PRV-GD2013.

**Conclusions:**

The PRV-GD2013-ΔgI/gE vaccine strain could be effective for immunizing Chinese swine herds against the pseudorabies virus (PRV) strain.

**Supplementary Information:**

The online version contains supplementary material available at 10.1186/s12917-021-02861-6.

## Highlights

1. Bartha-K61 did not provide full protection against the virulent PRV strains in China.
2. The PRV mutant was generated by homologous recombination.
3. The PRV mutant was non-toxic and immunogenic for immunized piglets.
4. The PRV mutant provided effective protection against the PRV variant challenge.

## Background

Pseudorabies (PR), widely known as Aujeszky′s disease, is an infectious disease for most livestock and wild animals caused by the pseudorabies virus (PRV). Pigs are a natural host while a variety of other vertebrates such as cattle, sheep, dogs, cats, goats, and rabbits, can be infected [1–4]. PRV infection in pigs produces different clinical symptoms, namely neurological symptoms and high mortality in piglets, growth retardation and respiratory disorders in growing pigs, and reproductive failure such as miscarriage and stillbirth in pregnant sows [5].

PRV is a double-stranded linear DNA virus of around 145 kb that belongs to the *Alphaherpesvirinae* subfamily of the *Herpesviridae* family [6]. The genome of PRV can be divided into a unique short region (US, 9 kb), a unique long region (UL, 110 kb), a terminal repeat sequence (TRS), and an internal repeat sequence (IRS) [7]. PRV encodes 10 glycoproteins, which are classified as essential and non-essential based on whether they are required for viral replication [6, 7]. The gE glycoprotein is encoded by the US8 gene. Its 125th valine and 126th cysteine have an important influence on the biological function of the virus. Deletion of these two amino acids reduces the neurotoxicity of the virus without affecting its immunogenicity [8]. The gI and gE glycoproteins together form a heterodimer complex, which is present in the infected cell membrane and the viral envelope membrane, and participates in the invasion of the nervous system by the pseudorabies virus and its transmission process [9–11]. The original vaccine strain, Bartha-K61, contains a genetic deletion of the gI, gE, US9, and US2 genes and is used worldwide, resulting in effective control of PR [12–14]. Between the 1990s and 2010, only a few swine PR cases were reported [15]. However, since 2011, PR outbreaks have occurred in pigs that were immunized with Bartha-K61, leading to severe economic losses in China [14, 16–19]. Consequently, several gene mutant vaccines of gE, gI&gE, gE&TK, gI&gE&TK deletion from PR variant have been generated [20–23]. Furthermore, studies have found that the effective reproduction rate (which represents the average number of secondary infections resulting from a typically infected swine at a given time within a population in which not all hosts are necessarily susceptible) during each year for the variant PRV was higher than one during 2012 to 2017 in China. This indicates a high risk of a variant PRV epidemic and that the safety or immunogenicity of these mutant vaccines is far from being satisfactory [24].

The overlapping PCR technique was developed by Horton et al. in 1989 [25]. This technology utilizes PCR to carry out effective gene recombination and site-directed mutagenesis in vitro. It can rapidly link two or more DNA fragments together without affecting the non-target DNA sequences. This technology facilitates the correct assembly of multiple fragments and accelerates complex genetic engineering, such as the production of polyclonal antibodies, somatic cell knockout of human cells, vaccine research, and assembly of viral genomes [26].

Our preliminary studies showed that the PRV variant isolated from infected swine that were immunized with Bartha-K61 had high pathogenicity and mortality. More than 200 pig farms were infected with this virus in China, which caused direct economic losses of more than 200 million RMB during outbreaks. Biological characterization and sequence analysis confirmed that the isolated strain, hereafter referred to as PRV-GD2013, had many specific amino acid mutations when compared with different variants found in our previous study in China. Bartha-K61 was unable to provide sufficient protection against PRV-GD2013. Unfortunately, there is no effective vaccine on the domestic market that can control this PRV mutant. Therefore, there is an urgent need to develop a new vaccine that can control the PRV epidemic in pig farms. In this study, a gI/gE-deleted mutant was constructed by overlapping PCR and homologous recombination technology from a PRV strain PRV-GD2013, and the safety and immunogenicity of the PRV mutant were evaluated in piglets.

## Results

### Characterization of the gI/gE-deleted PRV mutant

**Fig. 1:**
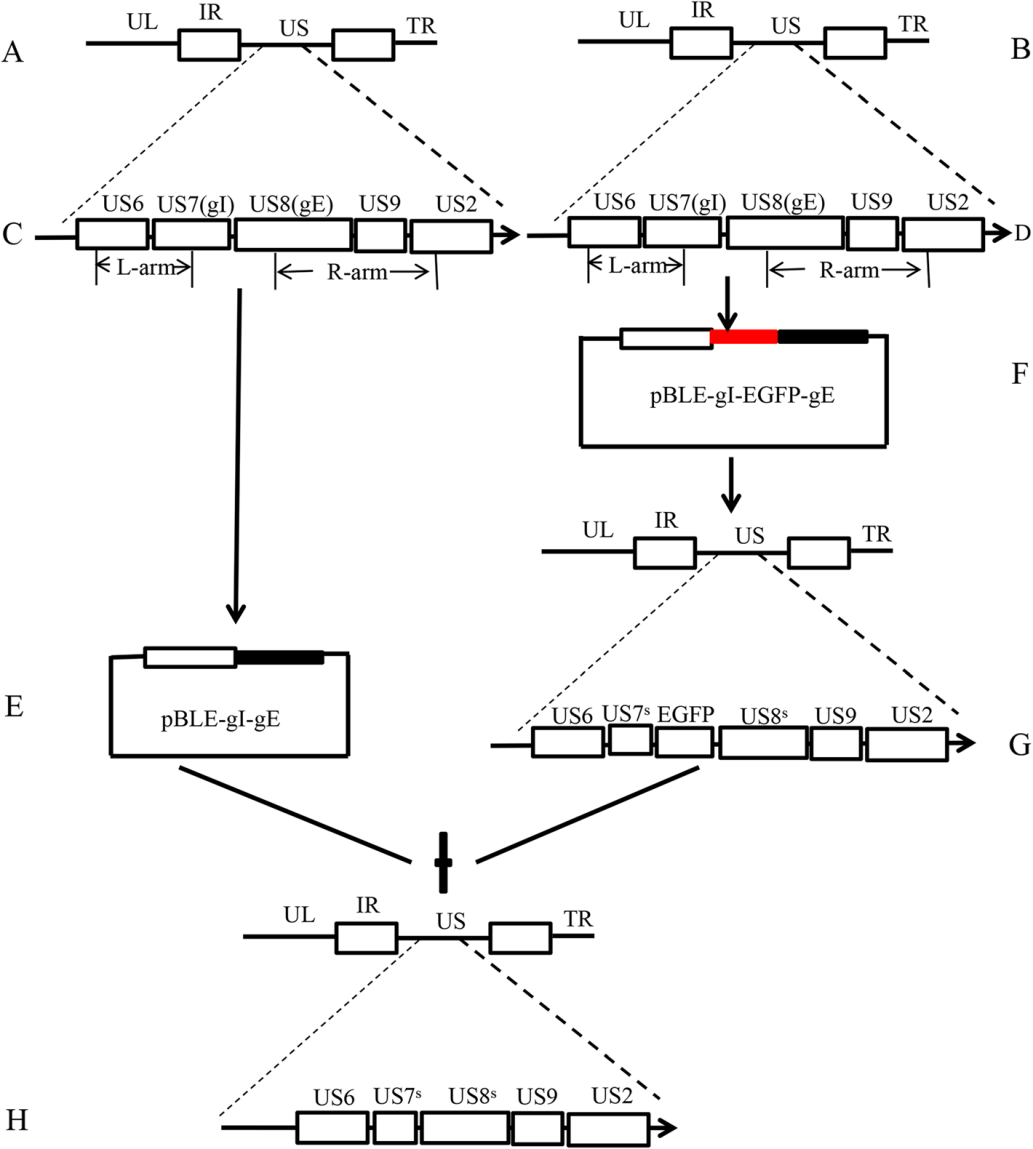
Construction strategy of the PRV-GD2013-ΔgI/gE recombinant strain. **a**, **b** Position of the left and right homologous recombination arms (L-arm and R-arm). **c**, **d** The genome of PRV-GD2013 and the relative site of US6 (gD), US7 (gI), US8 (gE), US9, and US2. **e** Construction of the transfer plasmids pBLE-gI-gE, including the target deletion region, L-arm, and R-arm. **f** Construction of the transfer plasmids pBLE-gI-EGFP-gE, including the target deletion region, L-arm, R-arm, and inserted EGFP expression cassette. **g** The genome of PRV-GD2013-ΔgI/gE-EGFP and the relative site of US6, US7s, the EGFP expression cassette, US8s, US9, and US2. **h** The genome of PRV-GD2013-ΔgI/gE and the relative site of US6, US7s, US8s, US9, and US2. UL, unique long region; US, unique short region; IR, internal repeat sequences; TR, terminal repeat sequences; US7s, section US7; US8s, section US8. EGFP expression cassette; EGFP (enhanced green fluorescent protein)

**Fig. 2:**
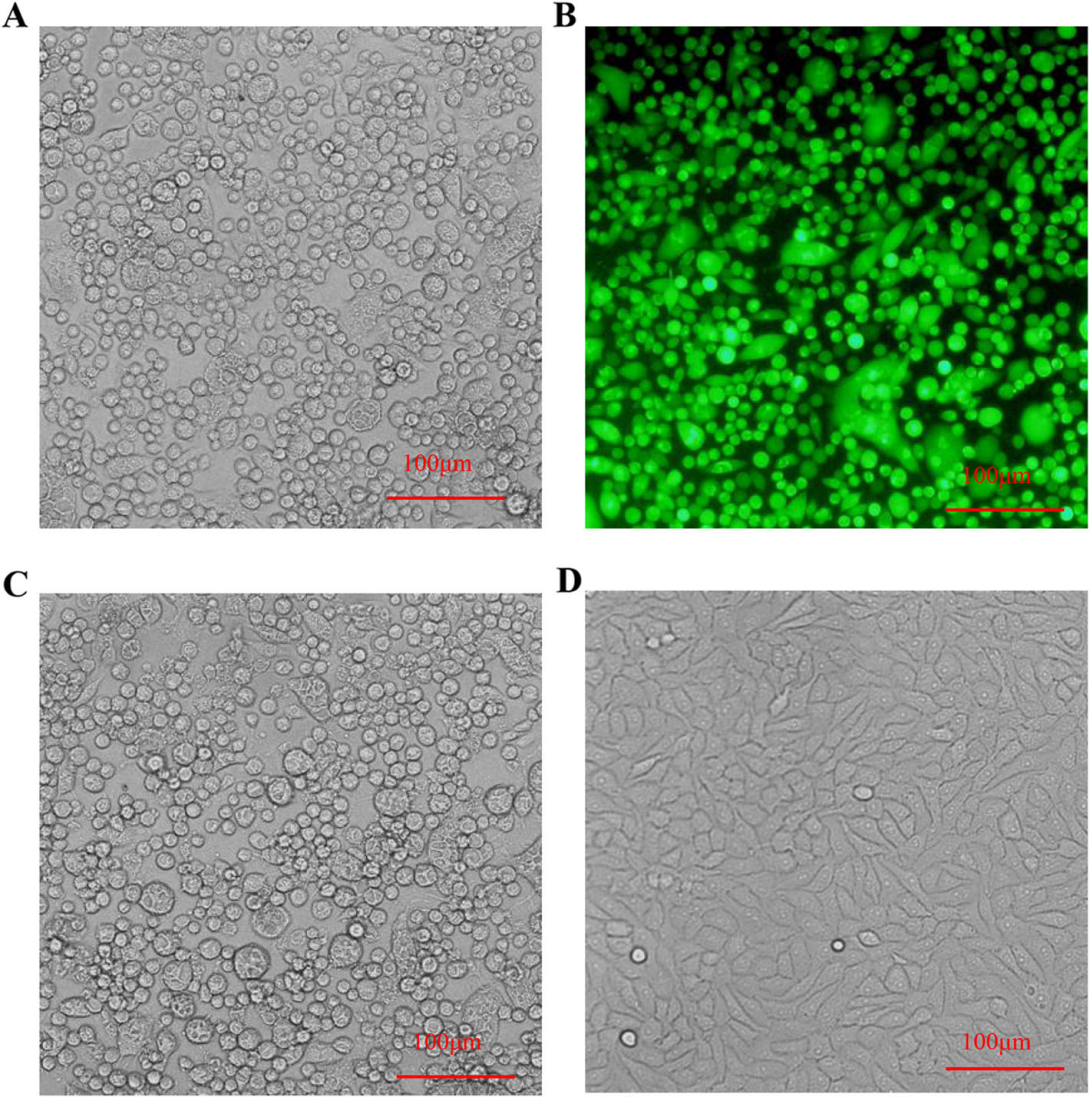
BHK-21 cells were infected with recombinant PRV. CPEs caused by PRV-GD2013-ΔgI/gE (**a**), PRV-GD2013-ΔgI/gE-EGFP (**b**), PRV-GD2013 (**c**), and control (**d**). CPEs were observed using a fluorescent microscope at two dpi

The pBLE (pBluescript SK (+)) and pBLE-EGFP (enhanced green fluorescent protein) vectors were used to construct pBLE-gI-gE and pBLE-gI-EGFP-gE vectors by overlapping PCR, and a recombinant PRV mutant was obtained in which gI/gE was deleted by homologous recombination (Fig. 1). After being subjected to five rounds of plaque purification under fluorescence microscopy, a gI/gE-deleted PRV mutant (PRV-GD2013-ΔgI/gE-EGFP) containing an EGFP gene was generated. Using the same method, a gI/gE-deleted PRV mutant (PRV-GD2013-ΔgI/gE) without an EGFP gene was produced. Lastly, the screening results were determined by fluorescence microscopy (Fig. 2). All the PRV-GD2013-ΔgI/gE infected cell without EGFP fluorescence compared to PRV-GD2013-ΔgI/gE-EGFP, which indicated that we successfully obtained the recombinant PRV-GD2013-ΔgI/gE (Fig. 2a, b). Recombinant viruses were analyzed by PCR (Fig. S1). The PCR results showed that the gene band size of line 1 was small when compared with lines 2 and 3, which indicated that we successfully deleted the gI/gE of PRV-GD2013, and, the DNA sequencing further verified this result (data not shown).

### Growth characteristics of the gI/gE-deleted recombinant virus

**Fig. 3:**
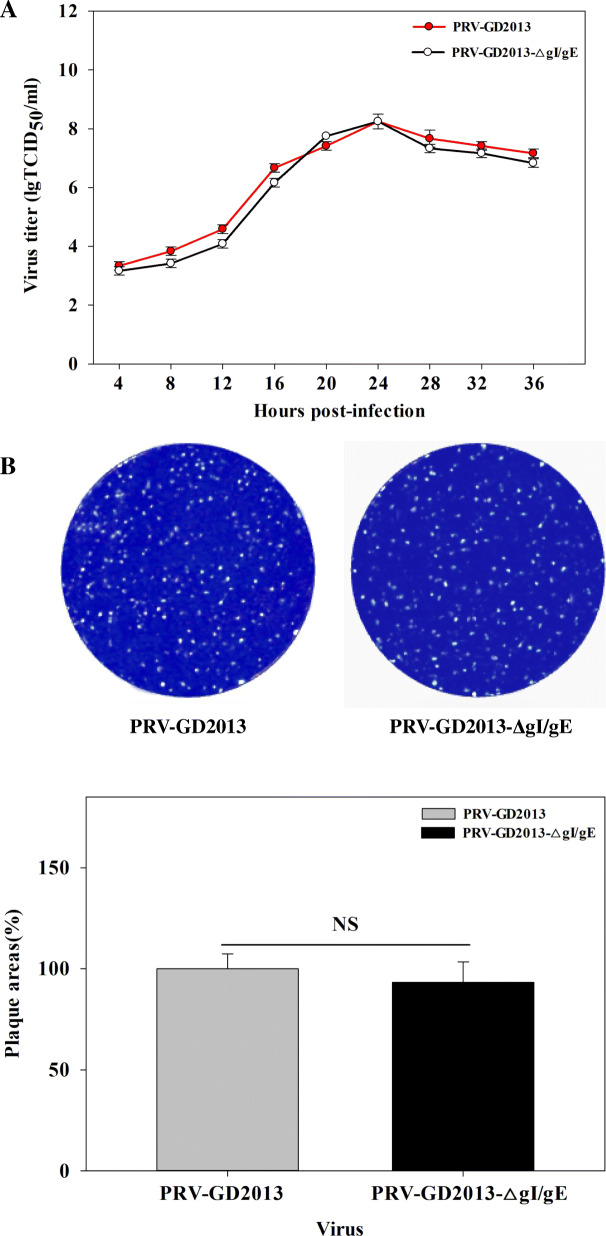
**a** One-step growth curve of PRV-GD2013-ΔgI/gE compared with its parental viruses. Monolayers of BHK-21 cells were inoculated with PRV-GD2013-ΔgI/gE and PRV-GD2013 at 1 MOI. The cell culture supernatants were collected at different time points (0, 4, 8, 12, 16, 20, 24, 28, 32, and 36 hpi), and were used to calculate the TCID_50_ of each virus. **b** Plaque morphology and plaque size measurement of PRV-GD2013-ΔgI/gE and PRV-GD2013 on BHK-21 cells at 60 h post-infection. The one-step growth curve and plaque size were measured by one-way repeated measurement analysis variance and least significance (LSD). Differences were considered statistically significant when *p* < 0.05

A one-step growth curve showed that there was no significant difference in the replication of PRV-GD2013-ΔgI/gE and the parental PRV-GD2013 strain in BHK-21 cells (*p < 0.05*) (Fig. 3a). After 24 h of infection, the peak titer of the recombinant virus was 10^8.0^–10^8.5^ TCID_50_/ml, which was the same as the parental strain. Meanwhile, the plaque areas between PRV-GD2013-ΔgI/gE reconstituted viruses and PRV-GD2013 were not significantly different (Fig. 3b).

### Virulence of the gI/gE-deleted recombinant virus in mice

Sixty mice were randomly divided into 12 groups of five and injected subcutaneously with different doses of PRV-GD2013 and PRV-GD2013-ΔgI/gE. The lethal doses (LD_50_) were 10^2.67^ TCID_50_ (PRV-GD2013) and 10^4.33^ TCID_50_ (PRV-GD2013-ΔgI/gE). The virulence of PRV-GD2013 in mice was higher than that of PRV-GD2013-ΔgI/gE, causing earlier death at the same dose (Table 1). The clinical symptoms of infected mice included pruritus at the injection site, biting of the skin, and hair loss.

**Table 1:** Outcome of infection with different doses of PRV-GD2013-ΔgI/gE and PRV-GD2013 in mice

Viruses	Groups	Amounts	Doses (TCID_50_)	Mortality (mean days to death)	LD_50_(TCID_50_)
PRV-GD2013	1	5	10^6.0^	5/5 (3.8)	10^2.67^
	2	5	10^5.0^	5/5 (4.0)	
	3	5	10^4.0^	5/5 (4.25)	
	4	5	10^3.0^	3/5 (5.0)	
	5	5	10^2.0^	1/5 (5.0)	
	6	5	10^1.0^	0/5	
PRV-GD2013-ΔgI/gE	7	5	10^6.0^	5/5 (5.0)	10^4.33^
	8	5	10^5.0^	5/5 (5.8)	
	9	5	10^4.0^	2/5 (6.0)	
	10	5	10^3.0^	1/5 (6.0)	
	11	5	10^2.0^	0/5	
	12	5	10^1.0^	0/5	
DMEM	13	5	0.1 ml	0/5	/

### Safety of the gI/gE-deleted recombinant virus in piglets

**Fig. 4:**
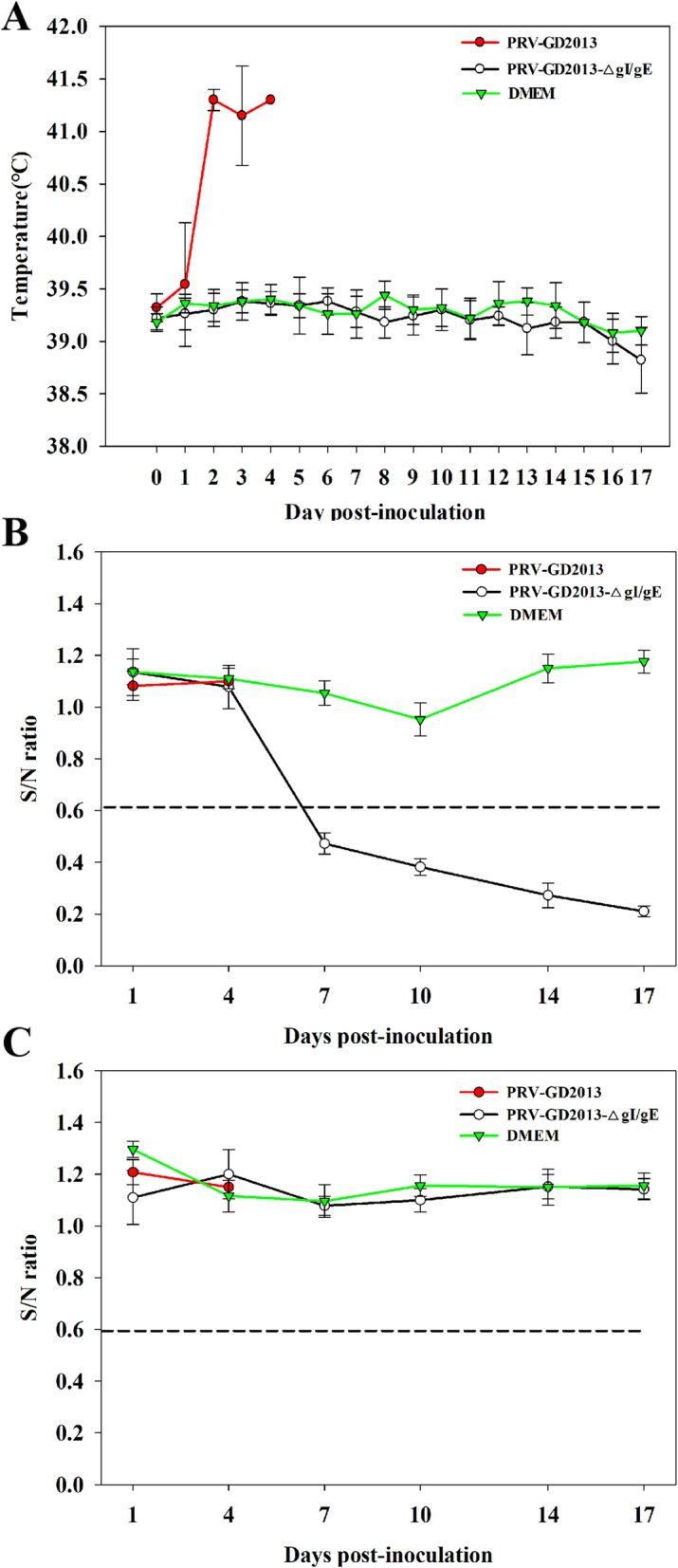
Pathogenic testing in piglets using recombinant PRVs. **a** Rectal temperature of piglets after inoculation with PRV-GD2013, PRV-GD2013-ΔgI/gE, or DMEM. **b** gB-specific antibody levels. Sample with S/N ratios ≤0.60, were classified as positive for gB antibodies. **c** Detection of gE-specific antibody levels. Samples with S/N ratios ≤0.60 were classified as positive for gE antibodies. All data are presented as the mean ± SD

**Fig. 5:**
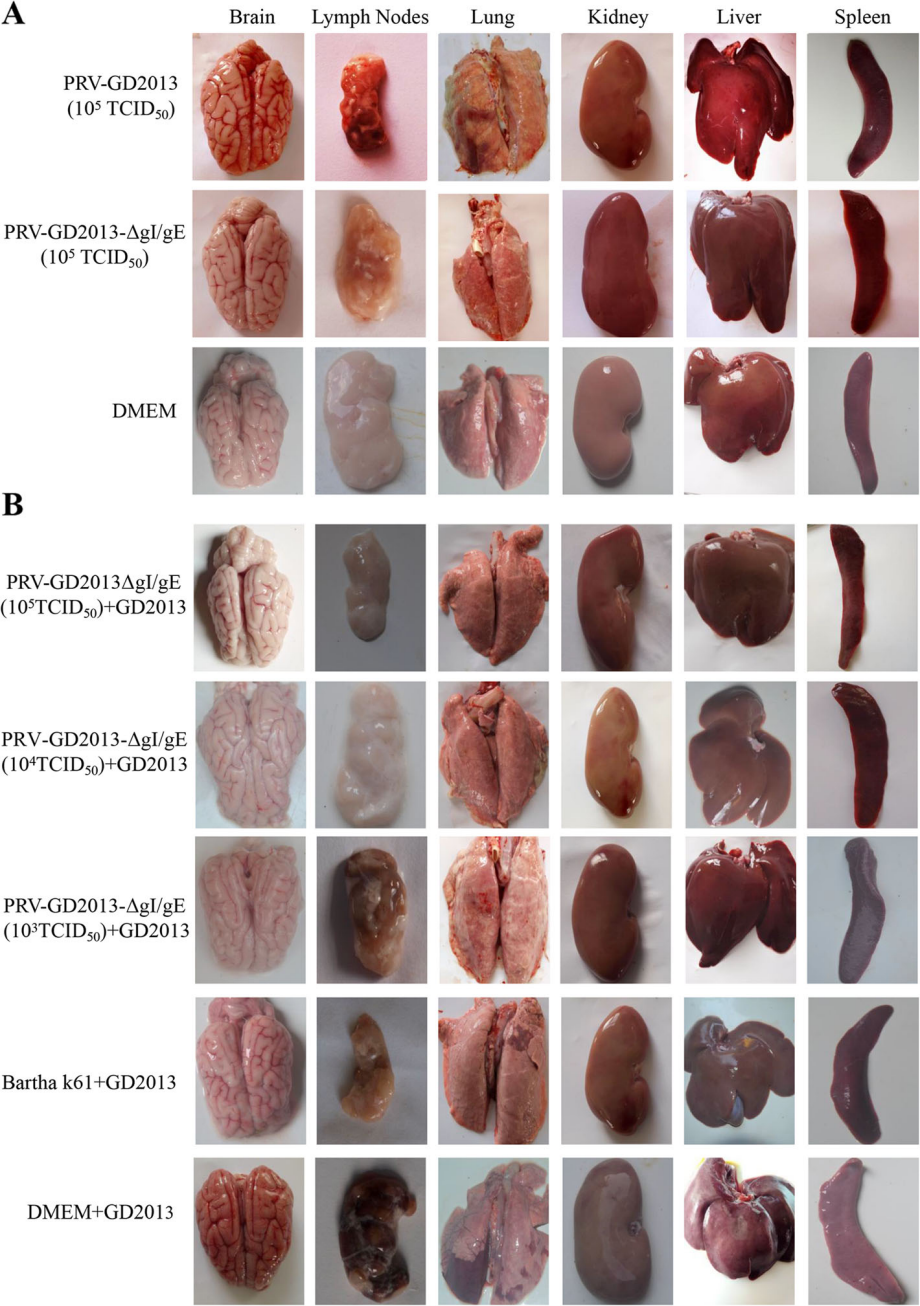
**a** Pathological examination of organ tissues. Groups of piglets (*n* = 3) were inoculated with 10^5^ TCID_50_ PRV-GD2013, 10^5^ TCID_50_ PRV-GD2013-ΔgI/gE, or DMEM. At 17 dpi, all surviving piglets were euthanized and necropsied. Tissue samples from the brain, lymph nodes, lung, kidney, liver, spleen were collected and used for pathological examination. **b** Pathological changes in various organ tissues of immunized piglets that were challenged with PRV-GD2013. Groups of piglets (*n* = 5) were inoculated with 10^5^ TCID_50_ PRV-GD2013-ΔgI/gE, 10^4^ TCID_50_ PRV-GD2013-ΔgI/gE, 10^3^ TCID_50_ PRV-GD2013-ΔgI/gE, Bartha-K61, or DMEM. At 14 dpc, all surviving piglets were euthanized and necropsied. Tissue samples from the brain, lymph nodes, lung, kidney, liver, spleen were collected and used for pathological examination

To evaluate the safety of gI/gE-deleted recombinant virus in piglets, fifteen 2-week-old piglets were randomly divided into 3 groups of five and inoculated with PRV-GD2013 (group A), 10^5^ TCID_50_ PRV-GD2013-ΔgI/gE (group B) and DMEM (group C) intranasally (i.n.). During the experiment, rectal temperatures, virus shedding, clinical symptoms and the level of gB/gE-specific antibodies were monitored and recorded. Piglets in experimental group A displayed a fever above 41 °C on the 2nd day after the infection, accompanied by mental depression, anorexia, respiratory distress, and neurological symptoms. Following inoculation, virus shedding was detected in all piglets and the first piglet died after 3 days, three died after 4 days, and the remaining piglet died after 5 days (Table 2). All piglets in groups B and C exhibited normal rectal temperatures, no challenge virus shedding was detected (Table 2) and all survived without showing any clinical symptoms during the experiment, which was a stark contrast to group A (Fig. 4a). No gB-specific antibodies were detected in all groups (Fig. 4b). However, the gE-specific antibodies were detected in group B, not in groups A and C (Fig. 4c). At 15 day post-inoculation (dpi) all surviving piglets in groups B and C were euthanized and necropsied. Necropsies of all of the piglets in group A showed obvious pathological lesions in the brain, lymph nodes, lung, kidney, liver, and spleen. Severe cerebral hemorrhage was the most obvious feature. However, the tissues and organs of piglets in groups B and C were normal and exhibited no obvious pathological changes when compared with the reactions of group A (Fig. 5a). These data indicate that the gI/gE-deleted recombinant virus was attenuated in piglets.

**Table 2:** Virus shedding data

Groups		Dose (TCID_50_)	Virus shedding day (post challenge)	Survival rate	Virus shedding
Pathogenic testing in piglets	(A)PRV-GD2013	10^5^	2 to death	0/5	5/5
	(B)PRV-GD2013-ΔgI/gE	10^5^	N	5/5	0/5
	(C)DMEM	2 ml	N	5/5	0/5
Immunization and challenge experiments	(A)PRV-GD2013-ΔgI/gE	10^5^	N	5/5	0/5
	(B)PRV-GD2013-ΔgI/gE	10^4^	N	5/5	0/5
	(C)PRV-GD2013-ΔgI/gE	10^3^	4–8	5/5	1/5
	(D)Bartha-K61	10^5^	3–9	5/5	4/5
	(E)DMEM	2 ml	2 to death	2/5	5/5

*N* None

### Protective efficacy of gI/gE-deleted recombinant virus in piglets

Twenty-five piglets were randomly divided into five groups of five and injected with 10^5^ PRV-GD2013-ΔgI/gE (group A), 10^4^ PRV-GD2013-ΔgI/gE (group B), 10^3^ PRV-GD2013-ΔgI/gE (group C), Bartha-K61 (group D), or DMEM (group E). Piglets in all groups exhibited normal rectal temperatures and no adverse clinical symptoms before the challenge. After 2 weeks of immunization, all of the piglets were infected with PRV-GD2013. All piglets in groups A and B survived with just a transient fever (40 ~ 41.5 °C), with no other clinical symptoms and virus shedding (Table 2). However, Piglets in groups C, D, and E were not completely protected when compared to groups A and B. In group C, piglets had only one occasion of elevated rectal temperatures (40.4 ~ 41.5 °C). One piglet showed virus shedding from the 4th to the 8th day after infection (Table 2) and displayed mild clinical symptoms including mental depression, anorexia, and respiratory distress. Piglets in group D had elevated rectal temperatures (40.4 ~ 41.5 °C) on two separate occasions. Four piglets exhibited virus shedding from the 3th to the 9th day after infection (Table 2), alongside displaying severe clinical symptoms including mental depression, anorexia, and respiratory distress, two of which showed obvious sequela (growth retardation and intermittent convulsion). All piglets in group E showed virus shedding (Table 2) and exhibited rectal temperatures over 41 °C twice post-challenge, which was accompanied by mental depression, anorexia, respiratory distress, and neurological symptoms. Three piglets in group E died, on the 4th, 5th, and 7th day respectively post-challenge (dpc).

**Fig. 6:**
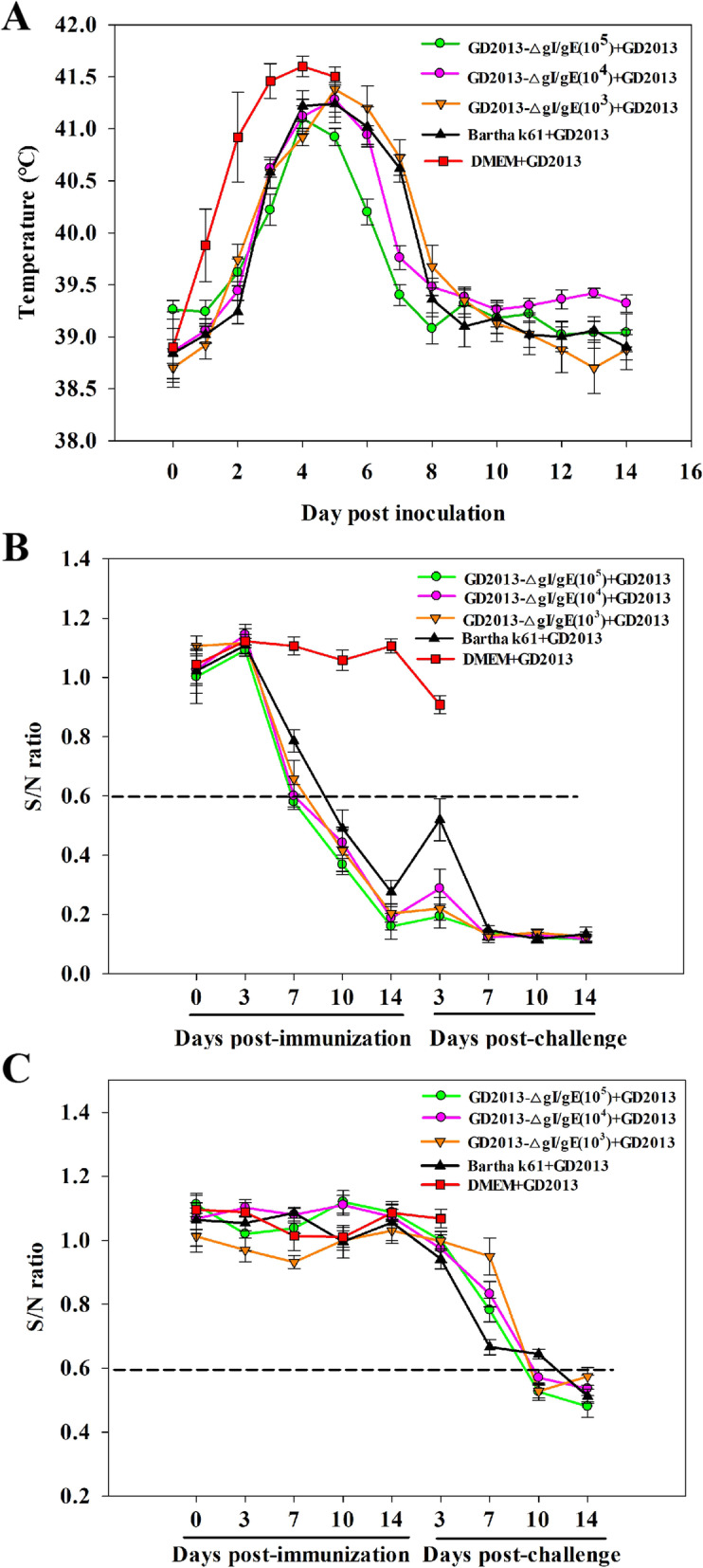
Immunization and challenge experiments in piglets. **a** Rectal temperature of piglets after challenge with PRV-GD2013. **b** gB-specific antibody levels. Samples with S/N ratios ≤0.60 were classified as positive for gB antibodies. **c** gE-specific antibody levels. Samples with S/N ratios ≤0.60 were classified as positive for gE antibodies. All data are presented as the mean ± SD

Serum samples taken from all piglets were analyzed for gB and gE-specific antibodies at 0, 3, 7, 10, 14 (0), 17 (3), 21 (7), 24 (10), and 28 (14) days post-immunization. The numbers in parentheses represent the number of days after the infection was injected. gB-specific antibodies in all of the immunized piglets in groups A, B, C, and D, were detected at 7, 7, 10, and 10 dpi respectively. Levels of gB-specific antibodies showed a slight short-term decrease at three dpc, and then gradually increased. No gE-specific antibodies were detected in any piglets before the challenge. Production of gE-specific antibodies was detected in groups A, B, and C at 10 dpc, after which, gE-specific antibody levels gradually increased. At 14 dpc, gE-specific antibodies were detected in group D piglets. However, no gB and gE-specific antibodies were detected in group E piglets at any point during the experiment when compared to groups A-D (Fig. 6b, c).

At 15 dpc, all surviving piglets were euthanized and then necropsied. The tissues and organs of piglets in groups A and B were normal and no obvious pathological changes were observed. The brains and livers of piglets in groups C and D showed slight hemorrhage and lung consolidation compared to groups A and B. However, the pathological features of the piglets in group C were milder than those of group D. All piglets in group E exhibited pathological changes in the brain, lymph nodes, lung, kidney, liver, spleen. Severe cerebral hemorrhage was the most obvious feature when compared to other groups (Fig. 5b).

**Fig. 7:**
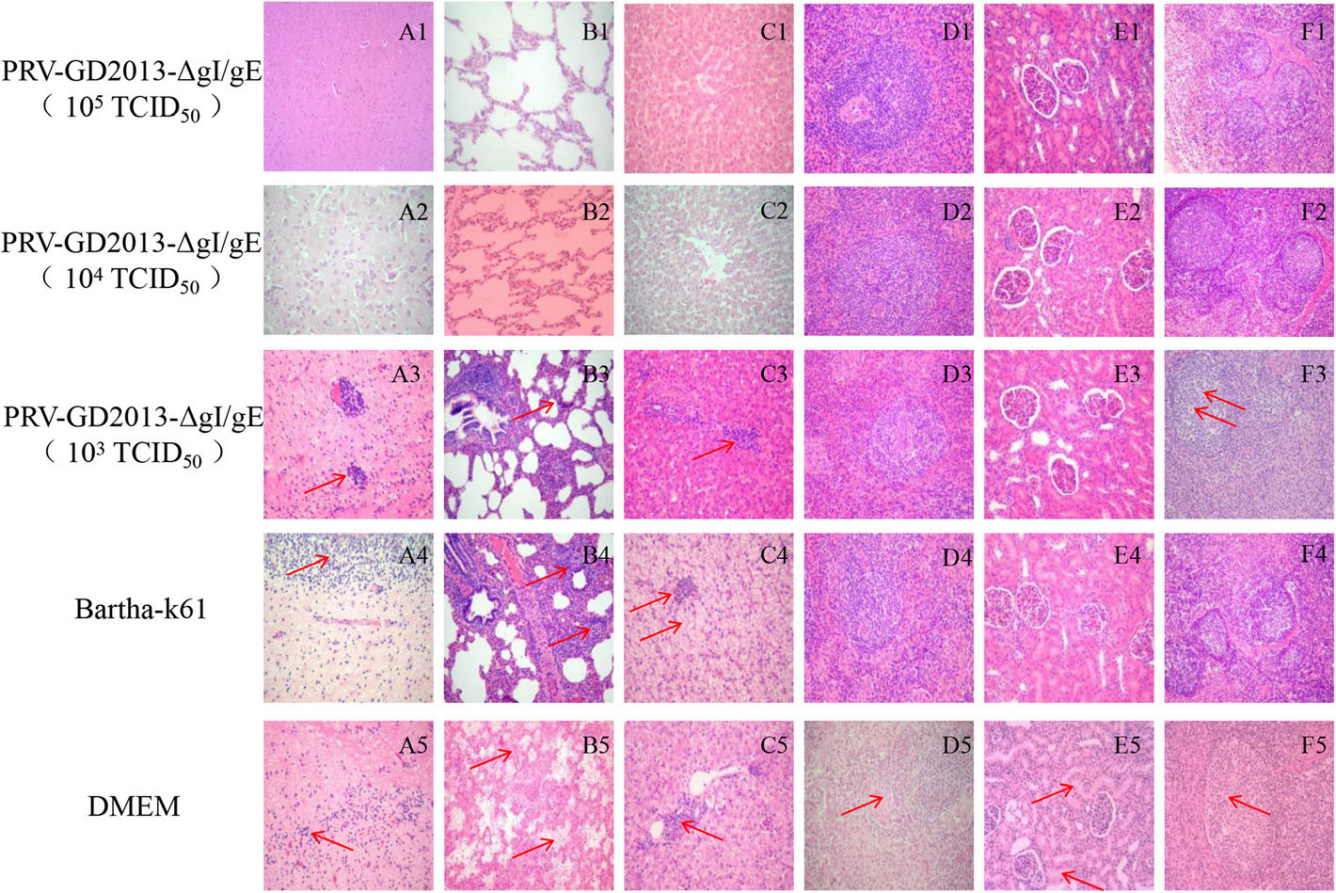
Histological examination of brain (**A1–A5**), lung (**B2-B5**), liver (**C2–C5**), spleen (**D2–D5**), kidney (**E2–E5**), and lymph nodes (**F2–F5**) of the piglets in different groups. **A1–F1** Correspond to piglets in 10^5^ TCID_50_ PRV-GD2013-ΔgI/gE vaccinated groups. **A2–F2** Correspond to piglets in 10^4^ TCID_50_ PRV-GD2013-ΔgI/gE vaccinated groups. **A3–F3** Correspond to piglets in 10^3^ TCID_50_ PRV-GD2013-ΔgI/gE vaccinated groups. **A4–F4** Correspond to piglets in Bartha k61 vaccinated groups. **A5–F5** Correspond to piglets in unvaccinated groups. Histopathologic examination and H&E staining. Magnification, 200 ×

Piglets immunized with 10^5^ TCID_50_ PRV-GD2013-ΔgI/gE and 10^4^ TCID_50_ PRV-GD2013-ΔgI/gE showed almost no obvious pathological lesions after challenged by PRV-GD2013. However, piglets immunized with 10^3^ TCID_50_ PRV-GD2013-ΔgI/gE, Bartha-K61, and DMEM showed different degrees of pathological lesions when compared to 10^5^ TCID_50_ PRV-GD2013-ΔgI/gE and 10^4^ TCID_50_ PRV-GD2013-ΔgI/gE group. Piglets immunized with 10^3^ TCID_50_ PRV-GD2013-ΔgI/gE exhibited some inflammatory cell infiltration in the brain, as well as alveolar wall thickening, hepatic portal area inflammatory cell infiltration, loose lymphocyte arrangement, and necrotic and disintegrated partial lymphocytes. The piglets in Bartha-K61 group also showed inflammatory cell infiltration in the brain, lung consolidation, inflammatory cell infiltration, lung abscesses, hemorrhage, and alveolar effusion, liver occasional punctate necrosis and hepatocyte cavitation. The piglets in the DMEM group all had obvious inflammatory cell infiltration in the brain, inflammatory cell infiltration, lung abscesses, hemorrhage, alveolar effusion, liver occasional punctate necrosis and hepatic portal area inflammatory cell infiltration, spleen hemorrhage, renal tubular epithelial cell degeneration, necrosis, loose lymphocyte arrangement, necrotic and disintegrated partial lymphocytes. The pathological lesions in the DMEM group were significantly more severe than those in Bartha-K61 group (Fig. 7).

## Discussion

In the 1960s, PRV was found to be endemic in pig farms in China. Vaccination was considered to be an effective strategy to control and eradicate PRV. At present, Bartha-K61 is a commonly used vaccine in China with a good safety record, good immunogenicity, and effective protection. However, since 2011, Chinese pigs vaccinated with Bartha-K61 have been showing typical symptoms of PR, which is strongly infectious to piglets leading to a 50% mortality rate [14, 16]. Furthermore, the PRV-GD2013 strain was isolated from Bartha-K61-vaccinated pig herds, indicating that Bartha-K61 vaccine has limited protection against the PRV variants found in China. Since 2017, some variant PRVs have been detected in the eastern provinces of China [27]. Interestingly, there is a close relationship between PRV and geographical location [27]. Thus, there may be a need for the development of new vaccines in different regions of China. The GD2013 strain was compared with other PRV variants. Genetically, the GD2013 strain is closely related to the strains isolated in China in recent years, but it is relatively distant from isolates of western countries, including Becker and Kaplan. Additionally, when compared with the Classic SC strain, there are nucleotide insertions and mutations in the gE gene of the GD2013 strain, and, when compared with the JS-2012-China-2012, QXY-China-2013 and HeN1-China-2014, which has 8, 12 and 9 amino acid differences (Fig. S2). Thus, the newly emerging GD2013 strain is a PRV variant with unique molecular characteristics. However, the mechanism of how the variant evolved is unknown. Based on these findings, the development of a live attenuated vaccine based on a novel PRV variant would be crucial for PRV control. In this study, we constructed and evaluated PRV-GD2013-ΔgI/gE, derived from PRV variant PRV-GD2013 as a live vaccine candidate.

A live vaccine candidate requires safety, broad protection, and immunogenicity. The criteria of target genes for constructing a live vaccine candidate are inessential for replication but is essential for virulence [28]. Extensive studies have shown that gI and gE perfectly meet these standards [13, 29–31]. gI/gE forms a complex and is responsible for neurotropism and reactivation. Our studies confirmed that the growth curve and plaque size of PRV-GD2013-ΔgI/gE was not significantly reduced when compared to that of PRV-GD2013. Also, the LD_50_ of PRV-GD2013 was higher than PRV-GD2013-ΔgI/gE in mice, indicating that the gI/gE genes are related to the virulence of PRV. PRV can infect pigs of all ages and the mortality rate of suckling piglets can be as high as 100% [5]. The efficacy evaluation demonstrated that a single inoculation of 10^5^ TCID_50_ PRV-GD2013-ΔgI/gE was capable of providing full protection for piglets challenged with 10^5^ TCID_50_ PRV-GD2013. However, the DMEM and Bartha-K61 vaccinations did not provide full protection for piglets challenged with 10^5^ TCID_50_ PRV-GD2013. These results demonstrate that immunization with the PRV-GD2013-ΔgI/gE strain with a lower TCID_50_ affords sufficient protection for piglets challenged with PRV-GD2013.

Furthermore, in the growing pigs, the gB-specific antibodies generated by the PRV-GD2013-ΔgI/gE vaccine stimulated significant levels of the gB antibody against PRV-GD2013, whereas the commercial Bartha-K61 vaccine demonstrated substantially lower protection against PRV-GD2013, with the gE-specific antibodies not being detected throughout the experiment. These data further suggest that Bartha-K61 is insufficient for the prevention of PRV infection, but that the PRV-GD2013-ΔgI/gE strain is non-toxic to two-week-old piglets, and is immunogenic for immunized piglets.

## Conclusions

We generated a PRV-GD2013-ΔgI/gE PRV mutant with gI/gE deletion using overlapping PCR and homologous recombination techniques. The PRV-GD2013-ΔgI/gE strain is non-toxic to two-week-old piglets and has full immunogenicity against PRV-GD2013. Antibodies produced by PRV-GD2013-ΔgI/gE-immunized piglets can be distinguished from those produced by wild-type strain infection using commercial gE-ELISA and gB-ELISA kits. This technology can be used for differential diagnosis. We suggest that the recombinant virus PRV-GD2013-ΔgI/gE is an attractive vaccine candidate to control the current epidemic of swine PR in China.

## Methods

### Cells and viruses

The PRV strain GD2013 was isolated in 2013 from the brain tissue of a sick piglet that had been vaccinated with Bartha-K61 in Guangdong Province, China. All viruses were propagated in BHK-21 cells (ATCC), which were cultured in Dulbecco’s Modified Eagle’s medium (DMEM, Gibco) supplemented with 10% fetal bovine serum (FBS, Hyclone) at 37 °C in a humidified 5% CO_2_ incubator.

### Construction of transfer vector by overlapping PCR and homologous recombination technology

#### Construction of a pBLE-gI-gE transfer vector

DNA from the GD2013 strain was used as a template for PCR amplification using the primers listed in Table 3. The areas of the PRV genome flanking of gI and gE genes were amplified using primers P_1_/P_2_ and P_3_/P_4_, respectively. The amplified gene fragments were used as the recombination homologous arms (L-arm and R-arm) of the pBLE-gI-gE transfer vector. P_2_ and P_3_ contained the overlapping complementary sequences, while P_1_ and P_4_ directed cloning into the vector. Both products were gel-purified after the first round of PCR. The second round of PCR used the two resulting PCR products and the P_1_ and P_4_ primers to obtain the fragment encoding gI-gE. The fragments were then assembled by homologous recombination and ligated into NotI- and ClaI- cleaved pBluescript KS (+) vectors to generate the pBLE-gI-gE transfer vector (Fig. 1). The recombinant plasmid was confirmed by DNA sequencing. All primers used to amplify the sequence are listed in Table 3.

**Table 3:** Specific primer of homologous recombination arm used in prime Script 2step PCR

Primer	Sequence (5′-3′)
P_1_	5′- CGGGCTGCAGGAATTCGCGGCCGCCGAGTCGTGGCAGCTGACGCTGA −3′
P_2_	5′- ACGTCATCACGAAGGAGCCCAGGCAGCCGCGGAAGGCTTCGT −3′
P_3_	5′- ACGAAGCCTTCCGCGGCTGCCTGGGCTCCTTCGTGATGACGT − 3′
P_4_	5′- AGGTCGACGGTATCGATATCGATACTCGGTGAGCACCTTCCACA − 3′
P_1_′	5′- CGGGCTGCAGGAATTCGCGGCCGCCGAGTCGTGGCAGCTGACGCTGA − 3′
P_2_′	5′- CAGGGGATAACGCAGGAAAGAACATAGGCAGCCGCGGAAGGCTTCGT − 3’
P_3_′	5′- CAGCTCATTTTTTAACCAATAGGCCGGGCTCCTTCGTGATGACGT − 3’
P_4_′	5′- AGGTCGACGGTATCGATATCGATACTCGGTGAGCACCTTCCACA − 3’
P_5_′	5′- ACGAAGCCTTCCGCGGCTGCCTATGTTCTTTCCTGCGTTATCCCCTG − 3’
P_6_′	5′- ACGTCATCACGAAGGAGCCCGGCCTATTGGTTAAAAAATGAGCTG − 3’
gI-F	5′- GTCCGTAGCCTCCGCAGTACC − 3’
gI-R	5′- CTTCTGGCGCAGCTCGGCCAA − 3’
gE-F	5′- GTGACCACGGTGTGCTTC − 3’
gE-R	5′- ACAGCACGCAGAGCCAGA − 3’
gIF	5′- GTCCGTAGCCTCCGCAGTACC − 3’
gER	5′- AATGCGGGCGGACCGGTTC − 3’

#### Construction of the pBLE-gI-EGFP-gE transfer vector

The coding sequences flanking the gI and gE gene fragment were amplified using primers P_1_′/P_2_′ and P_3_′/P_4_′, respectively. The amplified gene fragments were used as the L-arm and R-arm of the pBLE-gI-gE transfer vector. A signal sequence fragment, CMV-EGFP-SV40 polyA, was determined from the pEGFP-C3 plasmid by PCR using primers P_5_′/P_6_′. The P_2_′/P_5_′ and P_3_′/P_6_′ primer pairs contained the overlapping complementary sequences, while P_1_′and P_4_′ contained NotI and ClaI recognition sites for directed cloning into the vector. Both products were gel-purified after the first round of PCR. The second round of PCR used the three resulting PCR products to obtain the fragment encoding gI–EGFP-gE. The fragments were then assembled by homologous recombination and ligated into NotI- and ClaI- cleaved pBluescript KS (+) vectors to construct the pBLE-gI-EGFP-gE transfer vector (Fig. 1). The recombinant plasmid was confirmed by DNA sequencing. All primers used to determine the sequence are listed in Table 3.

### Generation of recombinant viruses

The genomic DNA of PRV-GD2013 was extracted and purified using commercially available kits (AXYGEN, LOT#: AP-MIN-BF-VNA-250, USA) according to the manufacturer’s instructions. The pBLE-gI-EGFP-gE transfer plasmid and the PRV-GD2013 genomic DNA were co-transfected into BHK-21 (2 × 10^5^ cells/dish) cells using the Lipofectamine® 2000 transfection reagent (Invitrogen), according to the manufacturer’s instructions. After cytopathic effects (CPEs) were observed, the transfected culture was harvested. After two to three freeze-thaw cycles, monolayers of BHK-21 cells were inoculated with 100 μl of lysate per well and covered with 1% low-melting agarose. Recombinant viruses with green fluorescent plaques were screened under fluorescent microscopy. After several rounds of plaque purification, the recombinant virus stably expressing EGFP was screened and hereafter referred to as PRV-GD2013-ΔgI/gE-EGFP. Similarly, PRV-GD2013-ΔgI/gE-EGFP genomic DNA and pBLE-gI-gE were co-transfected into BHK-21 cells, in the same way, to generate a recombinant virus without EGFP expression, hereafter referred to as PRV-GD2013-△gI/gE (Fig. 1). Gene recombination was identified by PCR (using the primers were listed in Table 3, gI-F/gI-R, gE-F/gE-R, gIF/gER) and DNA sequencing.

### Growth kinetics

Growth kinetics were determined by using a one-step growth curve and plaque size calculation. Monolayers of BHK-21 cells were inoculated with PRV-GD2013-ΔgI/gE and PRV-GD2013 at an MOI of 1 respectively. At 2 hours post-infection (hpi), the monolayers were washed twice with phosphate-buffered saline (PBS), and 2 ml of DMEM (containing 2% FBS) was added. The culture supernatants (200 μl) were collected at different time points (0, 4, 8, 12, 16, 20, 24, 28, and 32 hpi). The supernatants collected at these nine-time points were then used to calculate the TCID_50_ of the virus according to the Reed-Muench formula. One-step growth curves were drawn based on the result of these calculations. Plaque sizes were performed for PRV-GD2013-ΔgI/gE and PRV-GD2013 as described previously [32].

### Animal experiments

#### Pathogenic testing in mice

Sixty-five two-week-old specific-pathogen-free (SPF) female BALB/c mice were obtained from the Laboratory Animal Center of Southern Medical University and were randomly divided into 13 groups of five. Groups 1–6 were subcutaneously injected in the inguinal region with 0.1 ml of different doses (10, 10^2^, 10^3^, 10^4^, 10^5^ or 10^6^ TCID_50_) of PRV-GD2013. Groups 7–12 were subcutaneously injected in the inguinal region with different doses (10, 10^2^, 10^3^, 10^4^, 10^5^ or 10^6^ TCID_50_) of PRV-GD2013-ΔgI/gE. The mice in group 13 were subcutaneously injected in the inguinal region with 0.1 ml of DMEM and served as negative controls. Clinical signs and mortality in the mice were observed and recorded daily after inoculation for 14 days. All surviving mice were euthanized by intravenous administration of an overdose of sodium pentobarbital after the experiment. The LD_50_ of the virus was calculated according to the Reed-Muench formula.

#### Pathogenic testing in piglets

Fifteen two-week-old piglets without PRV antibodies were obtained from a local farm and were randomly divided into three groups of five and housed separately. Group A was inoculated i.n. with 2 ml of 10^5^ TCID_50_ PRV-GD2013. Group B was inoculated i.n. with 2 ml of 10^5^ TCID_50_ PRV-GD2013-ΔgI/gE. Group C was inoculated i.n. with 2 ml of DMEM to serve as a negative control. Following inoculation, rectal temperatures and clinical symptoms were monitored and recorded daily. Virus shedding was determined by the daily collection of nasal and rectal swabs and blood samples were collected at 0, 3, 7, 10, 14, and 17 dpi. At 17 dpc, all surviving piglets were euthanized by intravenous administration of an overdose of sodium pentobarbital and were then necropsied within 2 h of death. Any piglets that died before the end of the experiments were immediately necropsied. Tissue samples were collected for pathological examination.

#### Immunization and challenge experiments

Twenty-five two-week-old piglets without PRV antibodies were obtained from a local farm and were randomly divided into five groups of five and housed separately. Groups A-C were vaccinated intramuscularly (i.m.) with different doses (10^5^, 10^4^, 10^3^ TCID_50_) of PRV-GD2013-ΔgI/gE. Group D was vaccinated i.m. with 10^5^ TCID_50_ Bartha-K61. Group E was inoculated i.m. with DMEM to serve as a negative control. After 2 weeks post-vaccination, all piglets were challenged i.n. with 10^5^ TCID_50_ of PRV-GD2013. After vaccination and challenge, rectal temperatures and clinical symptoms were monitored and recorded daily. Virus shedding was determined by the daily collection of nasal and rectal swabs, and blood samples were collected at 0, 3, 7, 10, and 14 dpi. At the end of the experiment, all surviving piglets were euthanized by intravenous administration of an overdose of sodium pentobarbital and were then necropsied within 2 h of death. Any piglets that died before the end of the animal experiments were immediately necropsied. Tissue samples from the brain, lung, liver, kidney, spleen, and lymph nodes were collected for histopathologic examination.

### Enzyme-linked immunosorbent assay (ELISA)

Levels of gB- and gE-specific antibodies were measured using commercial PRV antibody detection kits (IDEXX, USA) according to the manufacturer’s instructions.

### Statistical analysis

All experimental data were analyzed using SPSS 13.0 software and Sigma Plot 12.0. All data are presented as mean ± SD.

## Supplementary Information

**Additional file 1: Figure S1.** Recombinant viruses were analyzed by PCR. DNAMarker: DL5000; 1: PRV-GD2013-△gI/gE; 2: PRV-GD2013-△gI/gE-EGFP; 3: PRV-GD2013; 4: Negative control.

**Additional file 2: Figure S2.** Multiple alignment of the gE protein in PRV-GD2013, JS-2012-China-2012, QXY-China-2013 and HeN1-China-2014.

## Data Availability

The datasets generated and/or used during the current study are not available to public as it is owned by the South China Agricultural University, China. However, these can be requested via email from the corresponding authors; Mrs. Mingqiu Zhao (zmingqiu@scau.edu.cn) and/or Prof. Dr. Jinding Chen (jdchen@scau.edu.cn).

## Abbreviations

BHK-21: Baby Hamster Syrian Kidney
CPEs: Cytopathogenic effects
DNA: Deoxyribonucleic acid
dpc: Days post-challenge
dpi: Days post-inoculation
ELISA: Enzyme-linked immunosorbent assay
gB: Glycoprotein B
gC: Glycoprotein C
gD: Glycoprotein D
gE: Glycoprotein E
gG: Glycoprotein G
gI: Glycoprotein I
hpi: Hours post-infection
i.m.: Intramuscularly
i.n.: Intranasally
LD50: 50% lethal dose
MOI: Multiplicity of infection
PCR: Polymerase chain reaction
PR: Pseudorabies
PRV: Pseudorabies virus
TCID50: 50% tissue culture infectious dose
TK: Thymidine kinase
TRS: Terminal repeat sequence
IRS: Internal repeat sequence
US: Unique short region
UL: Unique long region
