# Supplementary figures and images for: Recombinant pseudorabies virus with gI/gE deletion generated by overlapping polymerase chain reaction and homologous recombination technology induces protection against the PRV variant PRV-GD2013
Source: BMC Vet Res. 2021 Apr 14;17:164. doi: 10.1186/s12917-021-02861-6 (PMC8048318; doi:10.1186/s12917-021-02861-6)

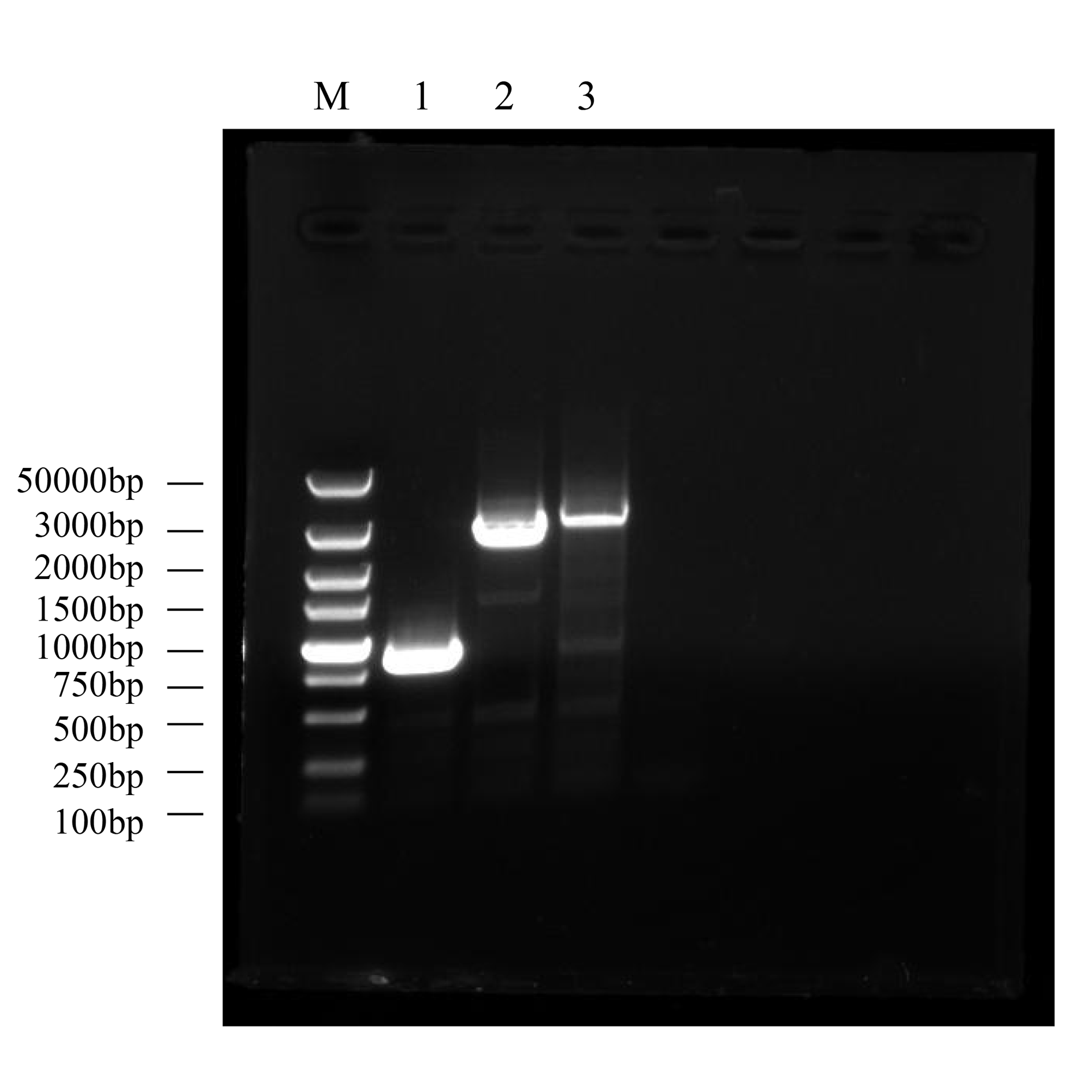

Supplement: Supplementary file 1 — Additional file 1: Figure S1. Recombinant viruses were analyzed by PCR. DNAMarker: DL5000; 1: PRV-GD2013-△gI/gE; 2: PRV-GD2013-△gI/gE-EGFP; 3: PRV-GD2013; 4: Negative control. [file 12917_2021_2861_MOESM1_ESM.tif]

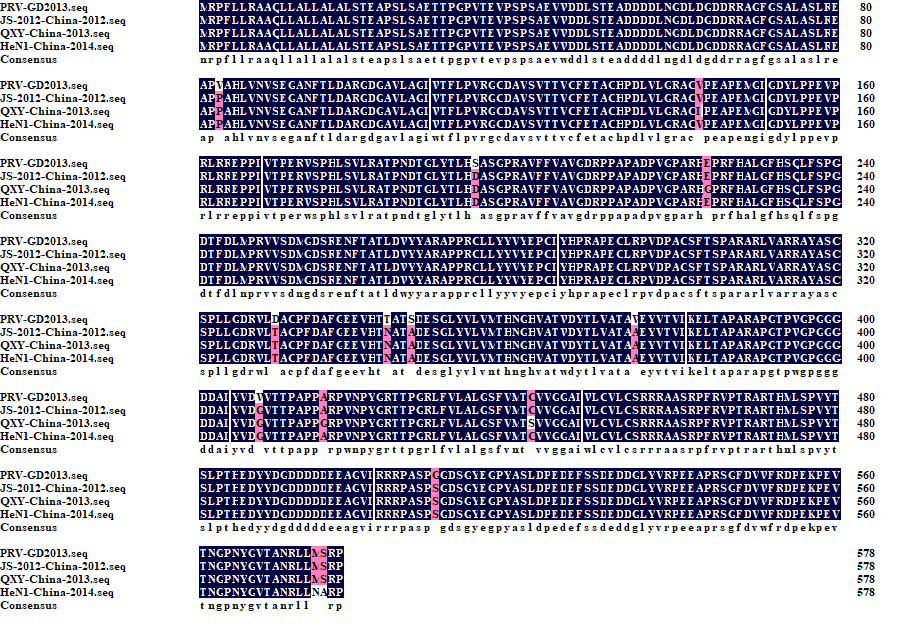

Supplement: Supplementary file 2 — Additional file 2: Figure S2. Multiple alignment of the gE protein in PRV-GD2013, JS-2012-China-2012, QXY-China-2013 and HeN1-China-2014. [file 12917_2021_2861_MOESM2_ESM.tif]
